# Terahertz Spectroscopy for Accurate Identification of *Panax quinquefolium* Basing on Nonconjugated 24(R)-Pseudoginsenoside F_11_

**DOI:** 10.34133/2021/6793457

**Published:** 2021-01-27

**Authors:** Tianyi Kou, Ji Ye, Jing Wang, Yan Peng, Zefang Wang, Chenjun Shi, Xu Wu, Xitian Hu, Haihong Chen, Ling Zhang, Xiaohong Chen, Yiming Zhu, Huiliang Li, Songlin Zhuang

**Affiliations:** ^1^Terahertz Technology Innovation Research Institute, Shanghai Key Lab of Modern Optical System, Terahertz Science Cooperative Innovation Center, University of Shanghai for Science and Technology, Shanghai Institute of Intelligent Science and Technology, Shanghai, China; ^2^Department of Pharmacy, Second Military Medical University, Shanghai, China; ^3^School of Pharmacy, Nanjing Medical University, Nanjing, China; ^4^Shanghai Institute of Intelligent Science and Technology, Tongji University Shanghai, China

## Abstract

*Panax quinquefolium* is a perennial herbaceous plant that contains many beneficial ginsenosides with diverse pharmacological effects. 24(R)-pseudoginsenoside F_11_ is specific to *P*. *quinquefolium*, a useful biomarker for distinguishing this species from other related plants. However, because of its nonconjugated property and the complexity of existing detection methods, this biomarker cannot be used as the identification standard. We herein present a stable 24(R)-pseudoginsenoside F_11_ fingerprint spectrum in the terahertz band, thereby proving that F_11_ can be detected and quantitatively analyzed via terahertz spectroscopy. We also analyzed the sample by high-performance liquid chromatography-triple quadrupole mass spectrometry. The difference between the normalized data for the two analytical methods was less than 5%. Furthermore, *P*. *quinquefolium* from different areas and other substances can be clearly distinguished based on these terahertz spectra with a standard principal component analysis. Our method is a fast, simple, and cost-effective approach for identifying and quantitatively analyzing *P*. *quinquefolium*.

## 1. Introduction

*Panax quinquefolium*, which is a herbaceous species in the family Araliaceae [1], is a valuable medicinal plant in many countries because of its antihypertensive, nerve cell stimulatory, antidiabetic, and anticancer properties. The medicinal qualities of *P*. *quinquefolium* are due to its diverse ginsenosides that are beneficial to humans, with extensive pharmacological effects on the central nervous system [2, 3], cardiovascular system [4, 5], endocrine system [6, 7], and immune system [8]. Moreover, these compounds are useful for treating cancer [9–11]. To date, more than 60 ginsenosides have been identified in *P*. *quinquefolium* [12]. According to the available information in the US and Chinese pharmacopeias, Rg_1_, Rb_1_, Re, and 24(R)-pseudoginsenoside F_11_ are the most important ginsenosides in *P*. *quinquefolium*. Similarly, Rg_1_, Rb_1_, and Re are the ginsenosides with the most important functions in ginseng. Thus, F_11_ is the main difference between *P*. *quinquefolium* and ginseng. Although *P*. *quinquefolium* and ginseng can be distinguished by traditional methods (e.g., analysis of appearance, application of microscopy, and examination of physical and chemical properties), high-performance liquid chromatography- (HPLC-) based mass spectrometry (MS) now represents the most authoritative and comprehensive method for differentiating between these species. For example, Wang et al. used HPLC-tandem MS (HPLC-MS/MS) and Chan et al. used HPLC-electrospray ionization MS (HPLC-ESI-MS) to distinguish *P*. *quinquefolium* from ginseng based on the distribution of ginsenosides [13, 14]. Ma et al. used HPLC-atmosphere pressure chemical ionization MS (HPLC-APCI-MS) to study the F_11_ content in *P*. *quinquefolium* and revealed that a majonoside isomer and the ginsenoside Rd may be used as markers to identify *P*. *quinquefolium* from China and North America [15]. However, these HPLC-based methods are currently useful only for laboratory research because they require complex pretreatment processes and they are costly and time-consuming [16]. Consequently, there is an urgent need for the development of a new, accurate, rapid, and qualitative and quantitative analytical method for identifying *P*. *quinquefolium* and the subsequent control of any derived drugs.

Researchers have confirmed that F_11_ is exclusive to *P*. *quinquefolium*, a unique biomarker for this species [17]. However, F_11_ is a nonconjugated substance that cannot be detected by a conventional HPLC-ultraviolet MS (HPLC-UV-MS) method. Moreover, most liquid chromatography methods cannot distinguish between F_11_ and the ginsenoside Rf because of their similar retention times [13, 14]. Thus, there is currently no available method for the rapid and accurate identification of F_11_.

The terahertz (THz) wave (0.1–10 THz) lying between the millimeter wave and the infrared band is of significant importance to the biological sciences because of complementary information to traditional spectroscopic measurements on low-frequency bond vibrations, hydrogen bond stretching, and bond torsions in liquids and gases [18]. Therefore, the collective behavior (vibration and rotation) characteristics of biomolecules make THz spectroscopy a promising sensing modality for clinical diagnosis [19]. Combining with the nondestructive, accurate, rapid, and good penetrability [20], THz spectroscopy also has many other potential applications in several research fields, including physics [21, 22], biology [23, 24], chemistry [25], and medicine [26].

In this study, we use THz spectroscopy to analyze F_11_ and *P*. *quinquefolium* and do the comparison with other methods. The detailed research contents are as follows: (I) calculate the theoretical vibration modes of F_11_ in the THz band; (II) experimentally confirm its characteristic THz absorption spectrum; (III) examine the THz absorption spectra of different *P*. *quinquefolium* samples; (IV) compare the quantitative data resulting from THz spectroscopic and HPLC-triple quadrupole-MS (HPLC-QQQ-MS) methods; and (V) distinguish *P*. *quinquefolium* from many similar substances based on the THz spectral characteristics of F_11_ via a principal component analysis (PCA). We herein describe a new method for the rapid qualitative and quantitative analysis of *P*. *quinquefolium*, which is expected to be extended to various plant detection.

## 2. Materials and Methods

### 2.1. Experimental Materials

The 24(R)-pseudoginsenoside F_11_ (>98%, CAS: 69884-00-0) and ginsenoside Re (>98%, CAS: 52286-59-6) used in this study were purchased from PureChem Standard (Chengdu, China). Polyethylene (PE) powder (particle size: 40–50 *μ*m, CAS: 9002-88-4) was purchased from Sigma-Aldrich (Shanghai, China). Additionally, 14 different batches of *P*. *quinquefolium* produced in North America and Jilin, China, were used in the form of block roots. All samples were used without further purification.

### 2.2. Sample Preparation for Terahertz Spectroscopy

Regarding the THz spectroscopy analysis, the sample preparation required grinding, sieving, and tablet pressing. Specifically, *P*. *quinquefolium* samples were ground to a powder with the MM400 ball mill (Retsch, Germany) at a vibration frequency of 90 Hz for 3 min. The powdered samples (particle size: 40–50 *μ*m) were placed under an infrared lamp to keep the sample dry, sieved, and mixed with PE powder (25% *w*/*w*) in an agate vessel. Samples were then compressed into tablets (1 mm thick and 13 mm diameter) with a hydraulic press (4 tons of pressure).

### 2.3. Sample Preparation for the HPLC Analysis

The *P*. *quinquefolium* crude powder (2.5 g) was added to a 100 mL round bottom flask, after which 25 mL methanol (analytical grade, 99.99%, CAS: 67-56-1; Fisher, USA) was added and the mixture was incubated overnight. The sample was heated under reflux in a 72°C water bath for 2 h, cooled, and filtered. The temperature was set according to the boiling point of methanol and the laboratory environment. Next, 25 mL methanol was added to the residue, after which the sample was heated under reflux for another 2 h, cooled, and then filtered with a qualitative filter paper (Hangzhou, China). The filtrate was added to a 50 mL volumetric flask, diluted with an equal volume of methanol, and thoroughly mixed before storing at −20°C [27, 28].

### 2.4. Sample Preparation for Additional Analyses

For UV spectroscopy, a 5 mg sample was dissolved in 1 mL water (analytical grade). The sample was diluted to 0.05 mg mL^−1^ prior to the analysis.

For Raman spectroscopy, the sample powder produced with the MM400 ball mill was placed on glass slides and analyzed with a Raman spectrometer.

For mid-infrared spectroscopy, the milled sample powder and KBr (Sigma-Aldrich, CAS: 7758-02-3) were mixed and compressed into tablets (1 mm thick and 13 mm diameter) with a hydraulic press (4 tons of pressure).

### 2.5. Experimental Instruments

The Terahertz experimental device is Brooke's Fourier transform infrared spectrometer (vectex80v, Bruker Optics). The far-infrared (IR) light source is a self-cooled mercury lamp, and the detector is a DLATGS/polyethylene detector. Therefore, the effective coverage of the spectral region is 30-680 cm^−1^, and the SNR is better than 10000 : 1. In the spectral range of 1.5 to 4 THz, with the resolution of 2 cm^−1^, the scanning times of 128, and the scanning speed of 5 kHz, all the spectra are measured at room temperature (~22°C) in a vacuum environment to reduce the influence of water vapor on the experiment.

The instrument used for HPLC results was Agilent 1200 high-performance liquid chromatography (MA, USA) and 6410 triple quadrupole mass spectrometer (MA, USA). The reagents used are acetonitrile and formic acid (chromatographic pure, Fisher, USA; content 88%, CAS: 64-18-6); methanol (chromatographic pure, Fisher, USA; content 99.99%, CAS: 67-56-1); ultrapure water (Milli-Q50 SP pure water system); and other reagents (analytic pure).

UV-VIS spectra were recorded using a UV-2450 spectrophotometer (Shimadzu, Japan). The test wavelength range is 190 nm-900 nm. Wavelength repetition accuracy is ±0.1 nm.

The Raman spectrometer model is DR-3168-LDC-DD (University of Shanghai for Science and Technology), The microscope in the machine is from Nikon (Japan). The spectral region is 400-3000 cm^−1^.

The MIR experimental device is Brooke's Fourier transform infrared spectrometer (vectex80v, Bruker Optics). The effective coverage of the spectral region is 400-7000 cm^−1^. In the spectral range of 400 to 4000 cm^−1^, we have the following: the resolution of 2 cm^−1^, the scanning times of 128, and the scanning speed of 5 kHz.

### 2.6. Absorption Peak Area Calculation

For the characteristic peak to be calculated, the slope of each point in the corresponding frequency range is calculated. When the slope of a point begins to be bigger than 0.3, it is regarded as the starting point of the region; when the slope of a point begins to be less than -0.3, it is regarded as the end point of the region. After determining the region range, the integral function of Origin software is used to do the area calculation.

### 2.7. Sample Preparation for Additional Analyses

The PCA refers to a statistical extraction method for simplifying datasets. It involves a linear transformation, with all principal components uncorrelated and ordered. Each principal component is a linear combination of the original variables. This transformation converts the data to a new coordinate system, with the largest variance on the first coordinate (i.e., first principal component (PC_1_)), the second-largest variance on the second coordinate (PC_2_), and so on (PC*_p_*) (Supplementary material 1) [29]. These PCs contribute the most to the variance in the dataset (e.g., material composition and spectral amplitude). Here, the PCA program we used is the function [COEFF SCORE latent] = princomp(x) built in MATLAB.

## 3. Results and Discussion

### 3.1. Theoretical Model

**Figure 1:**
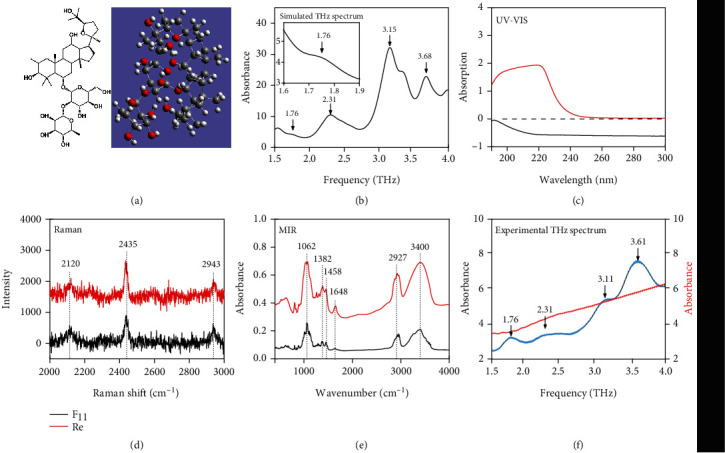
Different testing methods. Comparison for 24(R)-pseudoginsenoside F_11_. (a) Molecular formula and theoretical simulation model. (b) Theoretical simulation results. 24(R)-pseudoginsenoside F_11_ and ginsenoside Re (c) UV spectra, (d) Raman spectra, (e) mid-infrared spectra, and (f) terahertz spectra and the corresponding error bars (four tests).

24(R)-pseudoginsenoside F_11_, with a molecular formula of C_42_H_72_O_14_ and a molecular weight of 801.02 [30], is an ocotillol-type saponin (Figure 1(a)) [31, 32]. It is a dammarane-type triterpenoid that contains a four-*trans*-ring rigid steroid structure [33–35].

The molecular formula model (from the ChemSpider website, URL: http://www.chemspider.com) was imported into the GaussView quantum chemistry program [36], which applied the B3LYP hybrid functional with the 6-311G basis set. Additionally, the DFT-D dispersion correction was introduced.

### 3.2. Terahertz Spectroscopy Absorption Spectra

The molecular formula and simulation results for F_11_ (Figure 1(b)) revealed four characteristic absorption peaks at 1.76, 2.31, 3.15, and 3.68 THz. Analyses of the vibration/rotation of atoms/functional groups in the molecule indicated that the absorption peaks at 1.76 and 3.68 THz are mainly due to the wagging of the CH_3_ group, whereas the absorption peak at 2.31 THz is mainly the result of the wagging of the OH group in the hexatomic ring. The absorption peak at 3.15 THz represents the wagging of the CH_2_-OH group (Supplementary material 2).

We also used some traditional detection and comparison methods, including UV, mid-infrared, and Raman spectroscopic analyses. The nonconjugated F_11_ was compared with the ginsenoside Re, which is a conjugated biomarker of *P*. *quinquefolium*. Figure 1(c) presents the UV spectra of F_11_ and Re (0.05 mg mL^−1^). Because the *π* − *π*∗ transition wavelength range of nonconjugated systems is not within the stable operating range of the UV detector (190–700 nm), the presence of conjugated systems is determined based on the transition mode of organic compounds. In this study, Re was clearly identified as a molecule containing a conjugated system because of its obvious UV absorption peak at 220 nm. In contrast, a UV absorption peak was not detected for the nonconjugated F_11_. Thus, the UV spectrum was only able to determine the existence of a nonconjugated system but could not reveal its specific properties. The Raman spectra of F_11_ and Re (Figure 1(d)) included three peaks at 2120, 2435, and 2943 cm^−1^, corresponding to the polarization band of C ≡ C-C ≡ C symmetric stretching vibration and the antisymmetric stretching of CH_2_, with the same frequency and amplitude [37]. Raman spectroscopy is often used to examine functional groups. The presence of these three functional groups in F_11_ and other ginsenosides prevents them from being used to differentiate between ginsenoside compounds. In the mid-infrared spectra for F_11_ and Re (Figure 1(e)), peaks were detected at 1062 cm^−1^ (C-OH stretching vibration), 1382 cm^−1^ (CH_3_ symmetrical twisting vibration), 1458 cm^−1^ (CH_3_ asymmetrical twisting vibration), 1648 cm^−1^ (aromatic C-C stretching vibration), 2927 cm^−1^ (CH_2_ antisymmetric stretching vibration), and 3400 cm^−1^ (OH stretching vibration). Similar to the Raman spectroscopy results, the presence of the same peaks in the F_11_ and Re spectra indicated that the mid-infrared spectroscopy cannot be used for identifying ginsenosides. On the other side, the THz spectra of F_11_ and Re (Figure 1(f)) contained clear absorption peaks at 1.76, 2.31, 3.11, and 3.61 THz for F_11_ with a very small error bar, which are consistent with the theoretical data. In contrast, Re lacked an obvious absorption peak between 1.5 and 4.0 THz. The difference between Figure 1(e) and (f) is because the THz spectrum and mid-infrared spectrum have different frequency ranges, which correspond to different vibrational and rotational frequency characteristics of molecules. Here, the THz spectral fingerprint (low frequency) is more fit for the identification of the F_11_ and Re.

### 3.3. Comparison of Different Producing Areas

**Figure 2:**
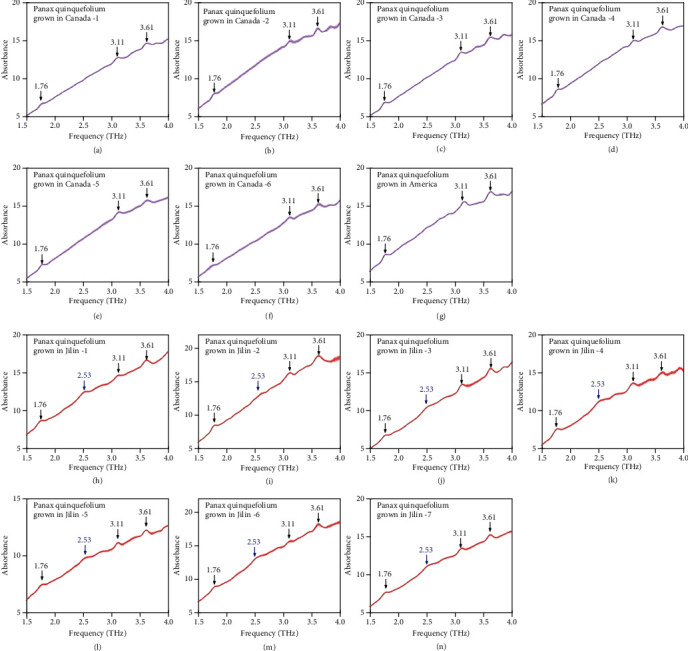
THz fingerprint spectra. Comparison for *Panax quinquefolium* from different producing areas. (a–g) THz spectra of North American *Panax quinquefolium* and the corresponding error bars (four tests). (h–n) THz spectra of Chinese (Jilin) *P*. *quinquefolium* and the corresponding error bars (four tests).

Because the growth of medicinal herbs is closely related to the natural environment (e.g., soil, water quality, and climate), their quality and efficacy vary among growing regions [38, 39]. Therefore, herbs from different locations need to be clearly distinguished and then used to produce specific medicines. In this study, we selected seven *P*. *quinquefolium* batches from North America (Canada and USA) and another seven batches from Changbai Mountain, Jilin province, China, to verify the utility of the F_11_ THz spectroscopy data for identifying *P*. *quinquefolium* harvested from different growing regions. For all *P*. *quinquefolium* samples, the F_11_ absorption peaks were detected, with the exception of the peak at 2.31 THz, which was too small (Figure 2). Moreover, the comparison between the North American and Chinese (Jilin) *P*. *quinquefolium* samples revealed an extra absorption peak at 2.53 THz for the Chinese batch. This peak may represent trace elements in the soil or reflect other climatic differences between the two examined growing regions [40]. So we can identify Chinese *P*. *Quinquefolium* and North American *P*. *quinquefolium* by using this additional absorption peak at 2.53 THz. We also used mid-infrared spectroscopy to do the producing area distinction (Supplementary material 3). However, the mid-infrared spectrum of *Panax quinquefolium* from different producing areas does not show stable differences in terms of the amplitude and position of absorption peaks. These results prove that the THz spectroscopy is more suitable and effective for identifying *P*. *quinquefolium* and for determining the source of the plant material.

### 3.4. Quantitative Analysis

To confirm the accuracy of our method, we analyzed samples by HPLC-QQQ-MS, which detected the F_11_ ions in the samples (i.e., ion peaks) (Supplementary material 4). This analysis was not influenced by the extent of the conjugation of a structure and was effective for detecting F_11_.

**Figure 3:**
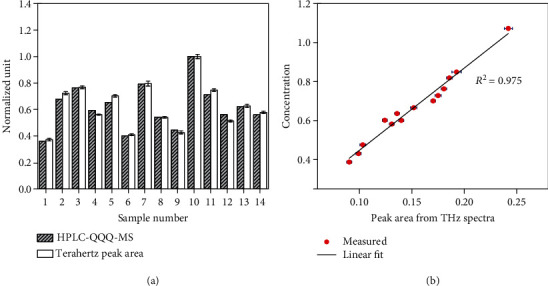
Quantitative analysis of *Panax quinquefolium*. (a) Histogram of the comparison between the normalized HPLC and THz spectroscopy data. (b) Analysis of the linear fit between the HPLC and THz spectroscopy data and the corresponding error bars.

In this experiment, 14 *P*. *quinquefolium* tablets were analyzed to ensure the consistency and comparability of the data. Regarding the THz spectra, the area of the absorption peaks represents the intensity of the molecular vibration (which is proportional to the concentration). Therefore, establishing the relationship between the peak area and the concentration enables the quantitative analysis of F_11_ in *P*. *quinquefolium*. The F_11_ concentration in *P*. *quinquefolium* was determined based on the HPLC-QQQ-MS data. A comparison between the normalized F_11_ concentration data derived from the HPLC-QQQ-MS analysis and the normalized peak areas of the corresponding samples in the THz spectra (Figure 3(a)) indicated that the relative difference between these two parameters was less than 5%. We subsequently established the relationship between the peak area and the F_11_ concentration (Figure 3(b)). The results revealed a linear relationship between the peak area and the F_11_ concentration (*R*^2^ = 0.975). Therefore, this linear relationship allows for the quantitative analysis of samples based on the peak areas. Because of the chemical loss associated with HPLC-QQQ-MS, we were unable to conduct multiple repetitions of this experiment. Consequently, the error bar and determination coefficient could not be determined. These results reflect the potential advantages of our THz spectroscopic method over other analytical methods (e.g., accurate, rapid, inexpensive, and no chemical losses).

To determine whether the THz spectrum of *P*. *quinquefolium* can be used to identify this species among similar and diverse materials, we performed a PCA to classify and identify samples. The analyzed materials included four batches of ginseng plants produced in 2016 and 2018, four kinds of non-Araliaceae herbs (white peony, red peony, Platycodon grandiflorum, and Atractylodes macrocephala), and 28 other substances (compound paracetamol tablet, morphine hydrochloride, glutamic acid, lysine, homocysteine, acetaminophen, caffeine, propyphenazone, phenylalanine, benzyl ester, amoxicillin, glimepiride tablet, saccharose, L-tryptophan, glucose, vitamin, aminobutyric acid, tyrosine, creatine, ammonium perchlorate, Intropin, cyclotetramethylenetetranitramine, PETN, trinitrotoluene, RDX (Hexogen), black powder, glucose lozenge, and amino acid lozenge). The corresponding THz spectra are presented in Supplementary material 5.

**Figure 4:**
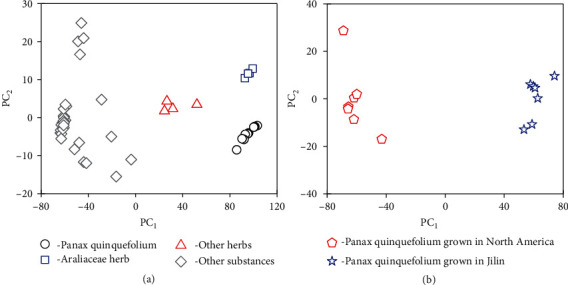
Principal component analysis based on terahertz spectra. (a) Principal component scores for 50 samples. Black circles, *Panax quinquefolium* samples; blue squares, ginseng from the family Araliaceae; red triangles, non-Araliaceae herbs; gray rhombi, other substances. (b) Principal component scores for *P*. *quinquefolium* from different growing regions. Red pentagons, seven batches of *P*. *quinquefolium* produced in North America; blue pentacles, seven batches of *P. quinquefolium* produced in China (Jilin).

Figure 4(a) presents the principal component scores for 50 samples based on the THz spectral data (PC_1_ = 98.4% and PC_1_ + PC_2_ = 99.4%). When the contribution rate of the first *p* principal components is large enough (PC_1_ + PC_2_ + ⋯+PC_*p*_ > 85%), the original dataset can be replaced approximately with the first *p* principal components [36]. Therefore, these spectral data can be analyzed approximately with PC_1_ and PC_2_. During the PCA, PC_1_ represents the largest differences between samples. The greater the PC_1_ value between two samples, the greater the difference between them [41]. The similarity in the PC_1_ scores for *P*. *quinquefolium* and ginseng (Figure 4(a)) is due to similarities in the contents of these Araliaceae herbs (e.g., Rb_1_, Rg_1_, and Re). The observed difference in the PC_2_ scores is caused by the F_11_ in *P*. *quinquefolium*. Additionally, the non-Araliaceae herbaceous plants had PC_1_ scores that were between those for the Araliaceae herbs and the 28 other substances. The substantial differences in the data for these 28 substances and the data for the Araliaceae herbs and herbaceous plants were explained by the fact that these other substances were derived from completely different species. Figure 4(b) provides the PC scores of *P*. *quinquefolium* produced in North America and China (PC_1_ + PC_2_ = 99.9%). The results indicated that in addition to being used to identify *P*. *quinquefolium*, our nonconjugated F_11_ detection method is also useful for distinguishing between *P*. *quinquefolium* samples collected from diverse growing regions.

## 4. Conclusion

In this study, we developed a new method enabling the accurate, rapid, and cost-effective identification and quantitative analysis of 24(R)-pseudoginsenoside F_11_. Our theoretical simulation and experimental data proved that F_11_ can be used as a biomarker for *P*. *quinquefolium*. Additionally, calculating the peak area can accurately quantify the F_11_ content in *P*. *quinquefolium* samples. A comparison between the THz spectroscopy and HPLC-QQQ-MS data indicated that the results differed by less than 5%, with a determination coefficient of 0.975. Finally, a PCA revealed that *P*. *quinquefolium* and other herbs or medicinal plants can be clearly distinguished, with PC_1_ useful for differentiating between *P*. *quinquefolium* plants from diverse growing regions.

We herein describe a new method for the qualitative and quantitative analyses of F_11_ in *P*. *quinquefolium* to differentiate between *P*. *quinquefolium* and other herbs or materials. In the future, this method combining THz energy enhancement or THz system signal-to-noise ratio improvement [19] and software analysis will pave the way for the identification of various plants, with potential commercial applications.

## Supplementary Materials

Supplementary Materials Supplementary 1 Principal component analysis method. Supplementary 2 Vibration mode analysis. Supplementary 3 Results of Panax quinquefolium MIR test. Supplementary 4 HPLC-QQQ-MS methods and results. Supplementary 5 THz spectra of substances used in PCA.
