# Supplementary material for: Terahertz Spectroscopy for Accurate Identification of Panax quinquefolium Basing on Nonconjugated 24(R)-Pseudoginsenoside F11
Source: Plant Phenomics. 2021 Jan 27;2021:6793457. doi: 10.34133/2021/6793457 (PMC8043154; doi:10.34133/2021/6793457)
Supplement: Supplementary Materials — Supplementary 1 Principal component analysis method. Supplementary 2 Vibration mode analysis. Supplementary 3 Results of Panax quinquefolium MIR test. Supplementary 4 HPLC-QQQ-MS methods and results. Supplementary 5 THz spectra of substances used in PCA. [file 6793457.f1.zip › Revised Supplementary_Material.docx]

Supplementary Material

**Supplementary 1: Principal component analysis method**

PCA is a statistical extraction method which reduces the dimensionality of a data set through mathematical transformation. The comprehensive variables derived from the transformation are the principal components which summarize the features of the data set. All the principal components are uncorrelated and ordered. Each principal component is a linear combination of the original variables.

PCA projects the n-dimensional features to the p-dimension (p<n). The greatest variance lies on the first coordinate, called the first principal component, the second greatest variance lies on the second coordinate, and so on. Consider a p-dimensional data matrix, X= (X_1_, X_2,_ …, X_p_ )^T^,

PC_1_ = a_1_’X = a_11_X_1_ + a_21_X_2_ + … + a_p1_X_p_

PC_2_ = a_2_’X = a_12_X_1_ + a_22_X_2_ + … + a_p2_X_p_

……

PC_p_ = a_p_’X = a_1p_X_1_ + a_2p_X_2_ + … + a_pp_X_p_

After the transformation, the original data of p variables X_1_, X_2_, …, X_p_ are replaced by the variables PC_1_, PC_2_, …, PC_p_ in the new coordinate system which successively inherit the maximum possible variance from X.

**Supplementary 2: Vibration mode analysis**

Supplementary Figure 1 shows the vibration modes of four terahertz absorption peaks (single molecule model), and the red dotted box region in the figure is the main vibration region. The frequency positions of absorption peaks in simulation result (Figure 1b) agree well with that of the experimental test results (Figure 1f).


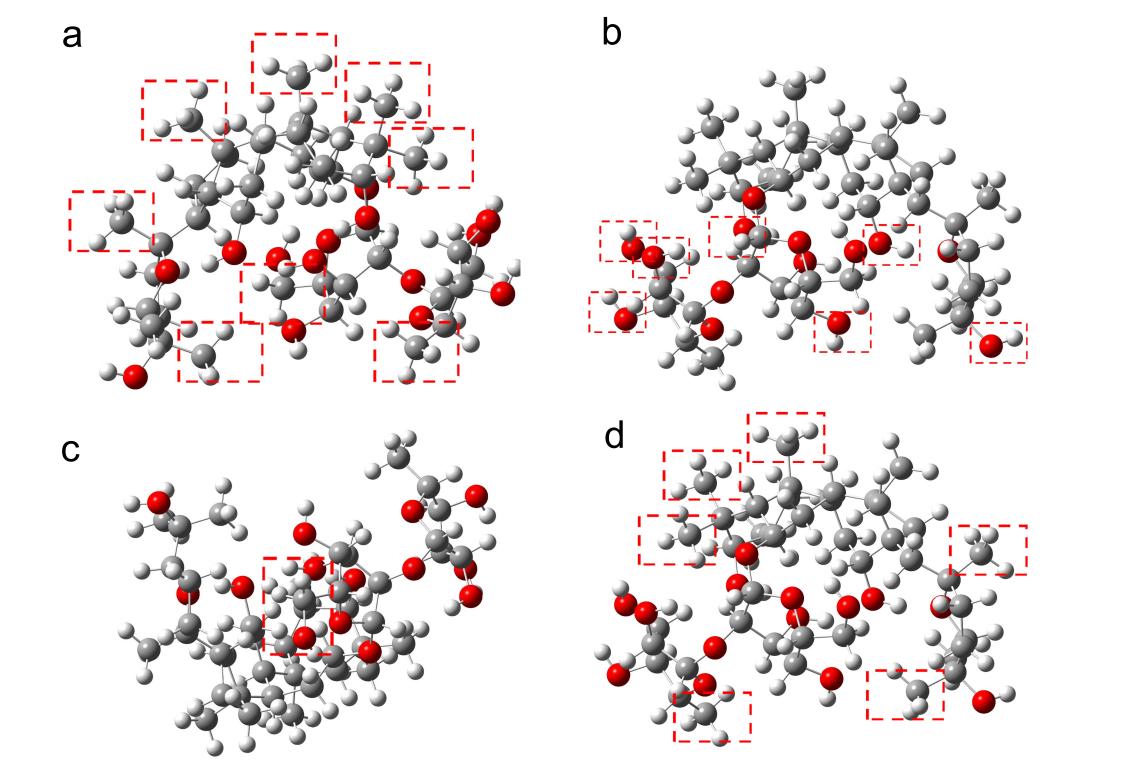


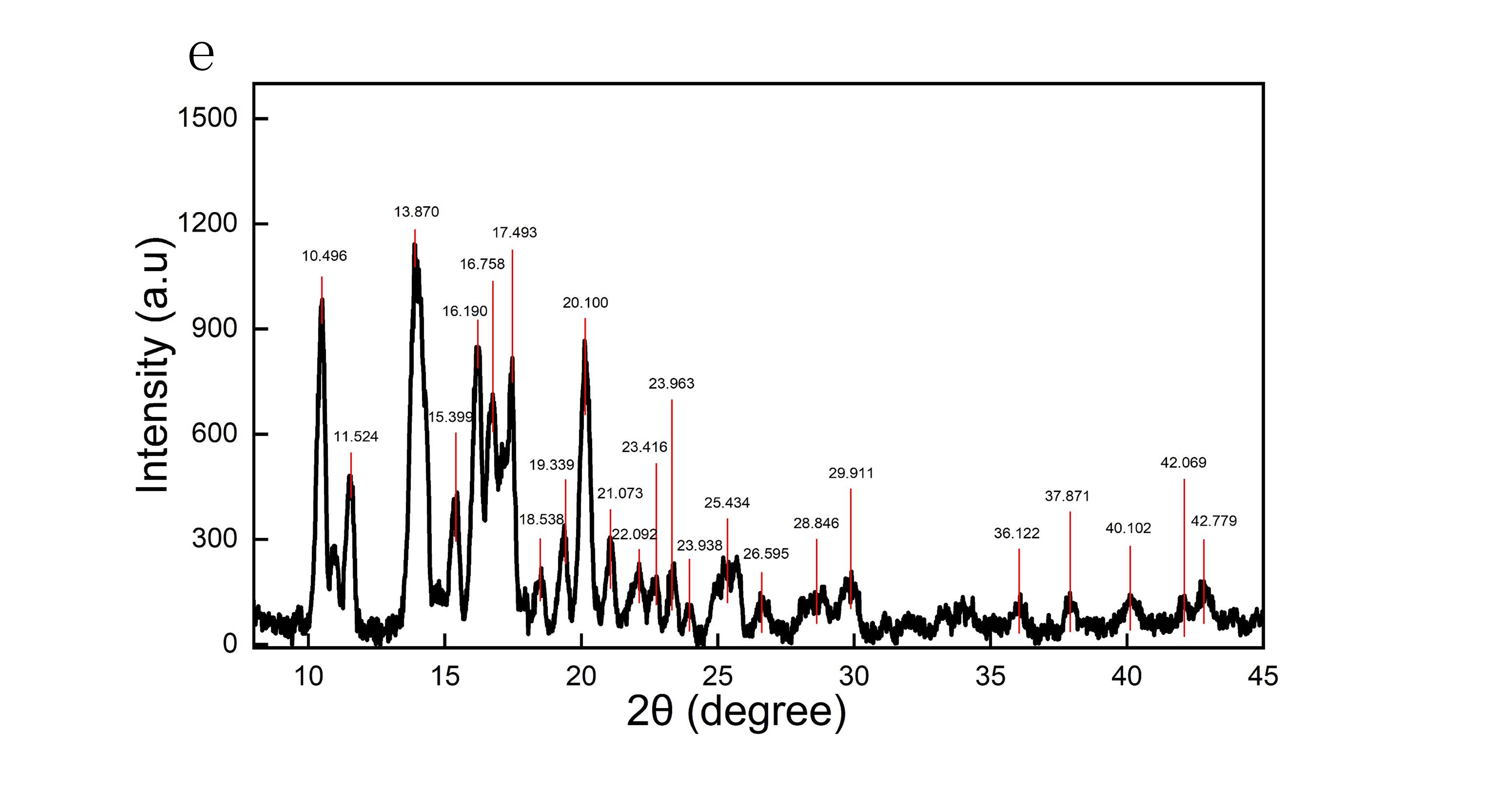


**Supplementary Figure 1.** **Vibration modes corresponding to four absorption peaks** **of F_11_ and XRD test.** (a) 1.76THz. (b) 2.31THz. (c) 3.15THz. (d) 3.68THz (e) XRD test result.

We also did the XRD test of the F_11_, and these peaks in Figure 1e show that F_11_ is a crystal structure at the molecular level. The intermolecular interaction must exist. It is just that in the experimental 1.5-4.0 THz range, the result of single molecular model is highly closed to the experimental test result. This may be attributed to the very large molecular weight of F_11_, the intramolecular interaction is complex and strong, and the intermolecular interaction doesn’t be reflected in this range of the spectrum.

**Supplementary 3: Results of *Panax quinquefolium* MIR test**

As shown in Supplementary Figure 2, the mid-infrared spectrum of *P. quinquefolium* from different producing area doesn’t show stable differences in terms of the amplitude and position of absorption peaks. Here, the mid-infrared spectral difference between F_11_ (Figure 1e) and *P. quinquefolium* (Supplementary Figure 2) may originate from the frequency shift and baseline caused by multiple intermolecular interactions. Therefore, this analysis using the mid-infrared spectroscopic information is complicated and not suitable. Compared with MIR, THz spectroscopy is relatively simple and intuitive, so we choose terahertz method for further study which fit for the producing area distinguish of *P. quinquefolium*.


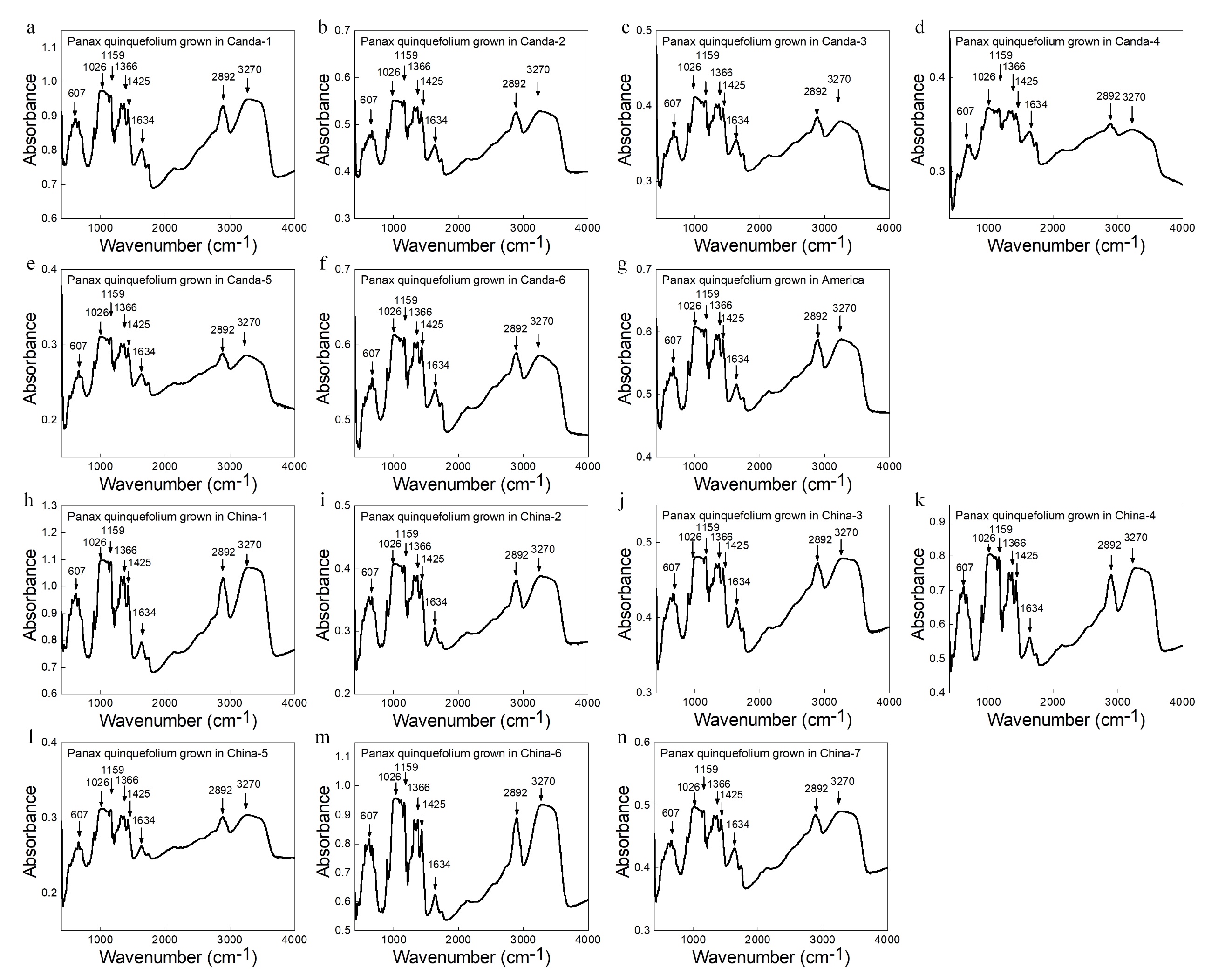


**Supplementary Figure 2.** **MIR tests for *Panax quinquefolium* from different producing areas.** (a) - (g) Chromatography of North America *P. quinquefolium*. (h) - (n) Chromatography of chinese Jilin *P. quinquefolium*.

**Supplementary 4: HPLC-QQQ-MS methods and results**

In our experiments, we used HPLC-QQQ-MS method as a comparison. The separation was performed on an Agilent Eclipse plus C18 column (3.0×150mm, 3.5 μm) at a column temperature of 30 °C and a flow rate of 0.4 ml min^-1^. The mobile phases consisted of water containing 0.1% formic acid (solvent A) and acetonitrile (solvent B), and elution occurred with linear gradient conditions as follows: 0–1 min, 28–28% B, 1-3 min, 28–70% B, 3–5 min, 70–100% B, 5-6 min, 100–28% B and 6-10 min, 28–28% B. The total run time was 10 minutes. Digoxin was induced as the internal standard during the quantification. Ionization was achieved using electrospray ionization in the negative ion mode with the capillary voltage set at 4000 V. Nitrogen was used as the nebulizer gas, and the nebulizer pressure was set at 40 psi with a source temperature of 105 °C. The desolvation gas (nitrogen) was set at 350 °C, and the drying gas flow rate was 10 L min^-1^. For collision induced dissociation (CID), high purity nitrogen was used as the collision gas at a pressure of 0.1 MPa. Multiple reaction monitoring (MRM) was employed for the quantitative analysis of F_11_, with the mass spectrometry parameters as following, MRM transitions (m z^-1^), 799.4→161.1, fragmentor, 275 V, collision energy, 34V, dwell time, 65 ms.

The standard stock solution was prepared by dissolving accurately weighed portions of F_11_ in methanol. The linearity study was achieved by diluting the stock solution to produce a series of concentrations. The calibration curves were constructed for at least six concentrations in triplicate. Calibration curve for F_11_ was constructed by plotting the integrated chromatography peak areas (Y) versus the corresponding concentration of the injected standard solution (X). Good linearity was observed, with *r*^2^>0.9995. The limit of detection (LOD) and limit of quantification (LOQ) were calculated by diluting the standard solution to when the signal to noise ratios (S/N) of the analytes were almost 3 and 10, and they were detected at 0.09 ng ml^-1^ and 0.42 ng ml^-1^, respectively. The relative standard deviation (RSD) values of one day and five consecutive days were shown to be within 2.27% and 3.17% respectively, which indicated that the precision of the experiment met the requirement. For the repeatability test, five independent working solutions were prepared from same batch using the same procedures noted in the “Sample preparation for HPLC test” section. The RSD value of repeatability was 4.59%. The RSD of stability was 4.02%, indicating the sample was stable within 24 hours. The average recovery rates were between 90.33% and 97.09% and the RSD value was 2.74%. These results indicated that the developed method is a reliable and useful method for determining the contents of F_11_. The quantitative results showed that the content of F_11_ with a mean content of 0.67 mg g^-1^ in *panax quinquefolium*.

Supplementary Figure 2 shows the chromatogram of the 14 samples, the peak time of F_11_ is ~5 minutes. The corresponding peak area are labelled on each peak.


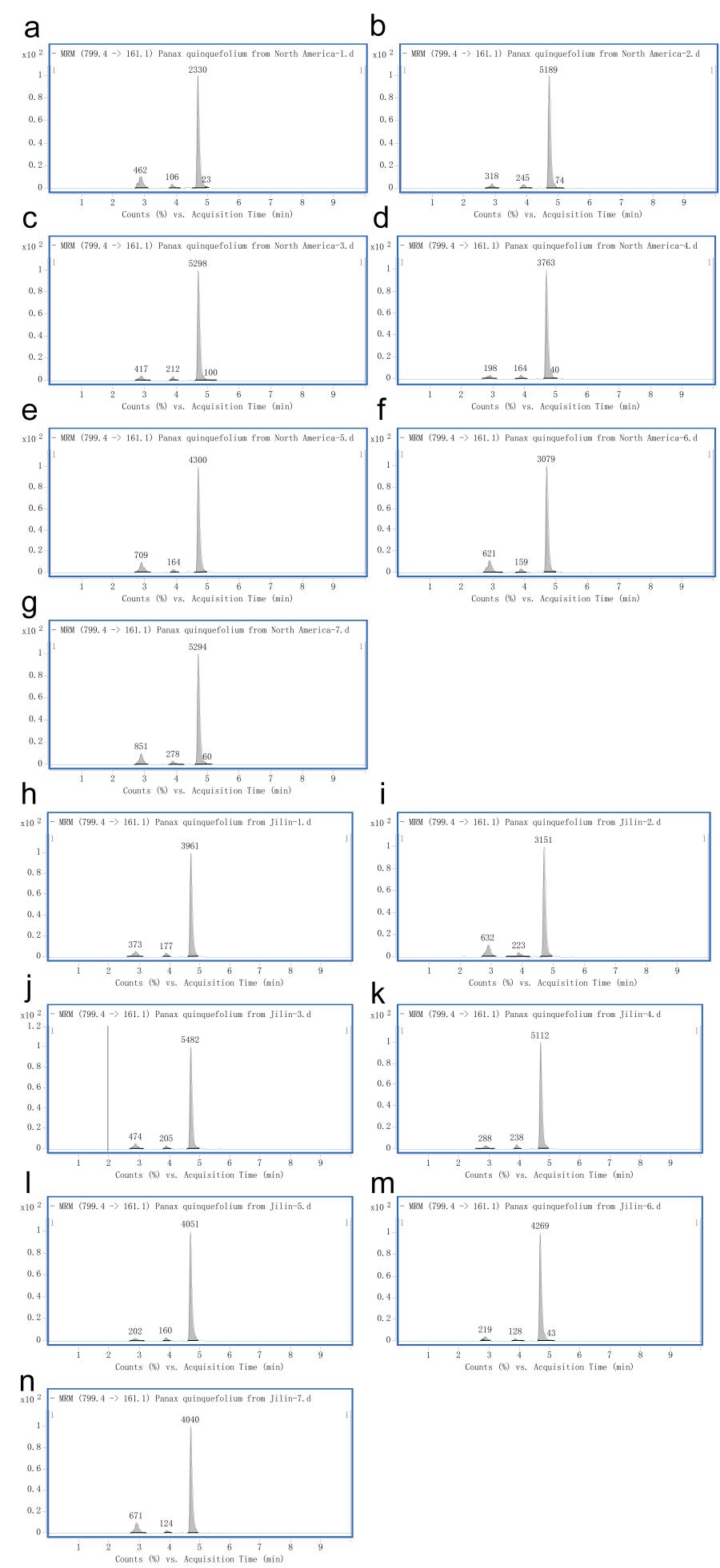


**Supplementary Figure 3.** **Chromatography tests for *Panax quinquefolium* from different producing areas.** (a) - (g) Chromatography of North America *panax quinquefolium*. (h) - (n) Chromatography of chinese Jilin *panax quinquefolium*.

**Supplementary 4:** **THz spectra of substances used in PCA analysis**

In the PCA analysis, we used Araliceae-ginseng, non-Araliceae herbs and other substances (such as western medicine, etc.) as qualitative controls for *panax quinquefolium*. Supplementary Figure 3. 4.5 shows the terahertz spectra of these samples.


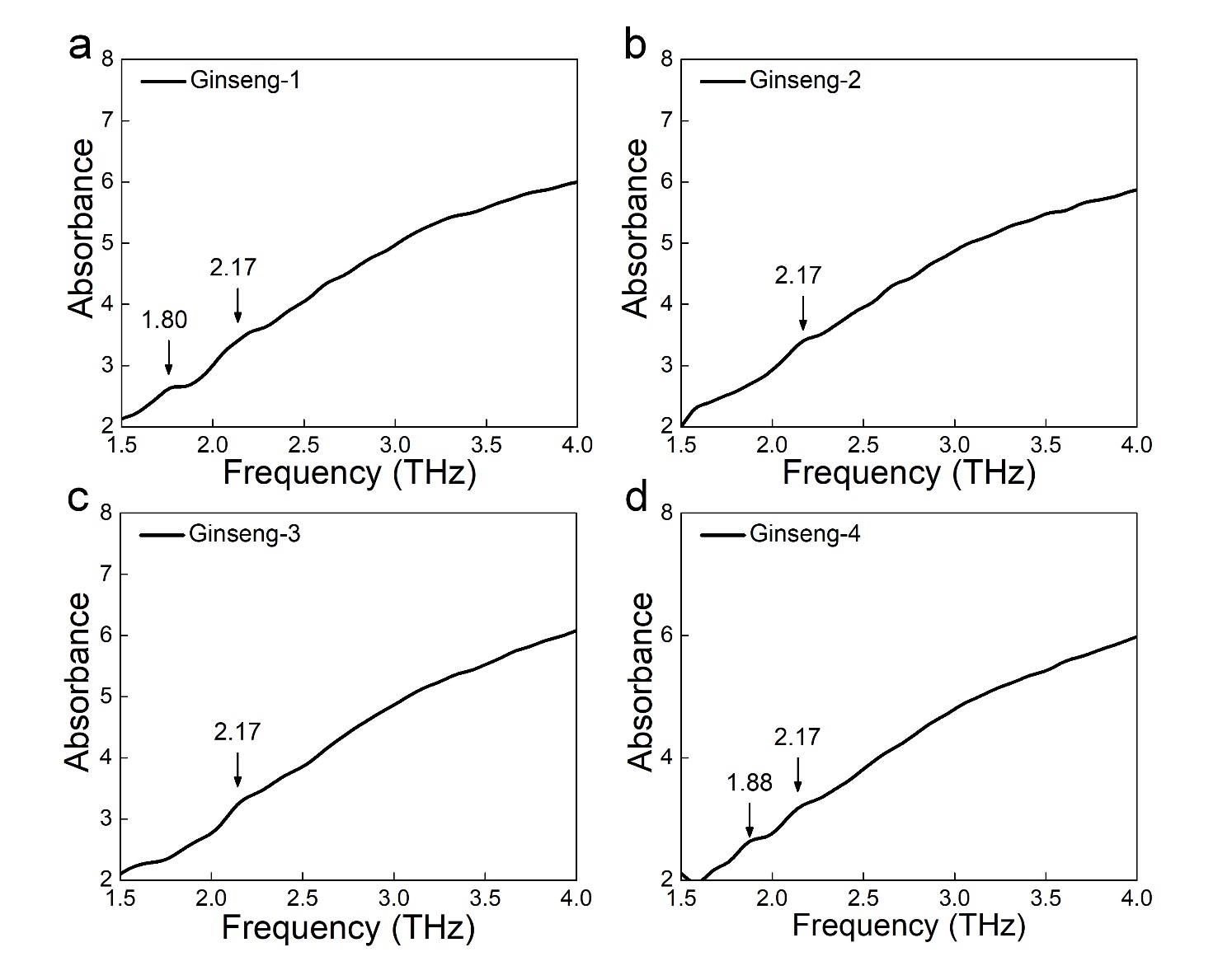


**Supplementary Figure 4.** **THz spectra of 4 batches ginseng.**


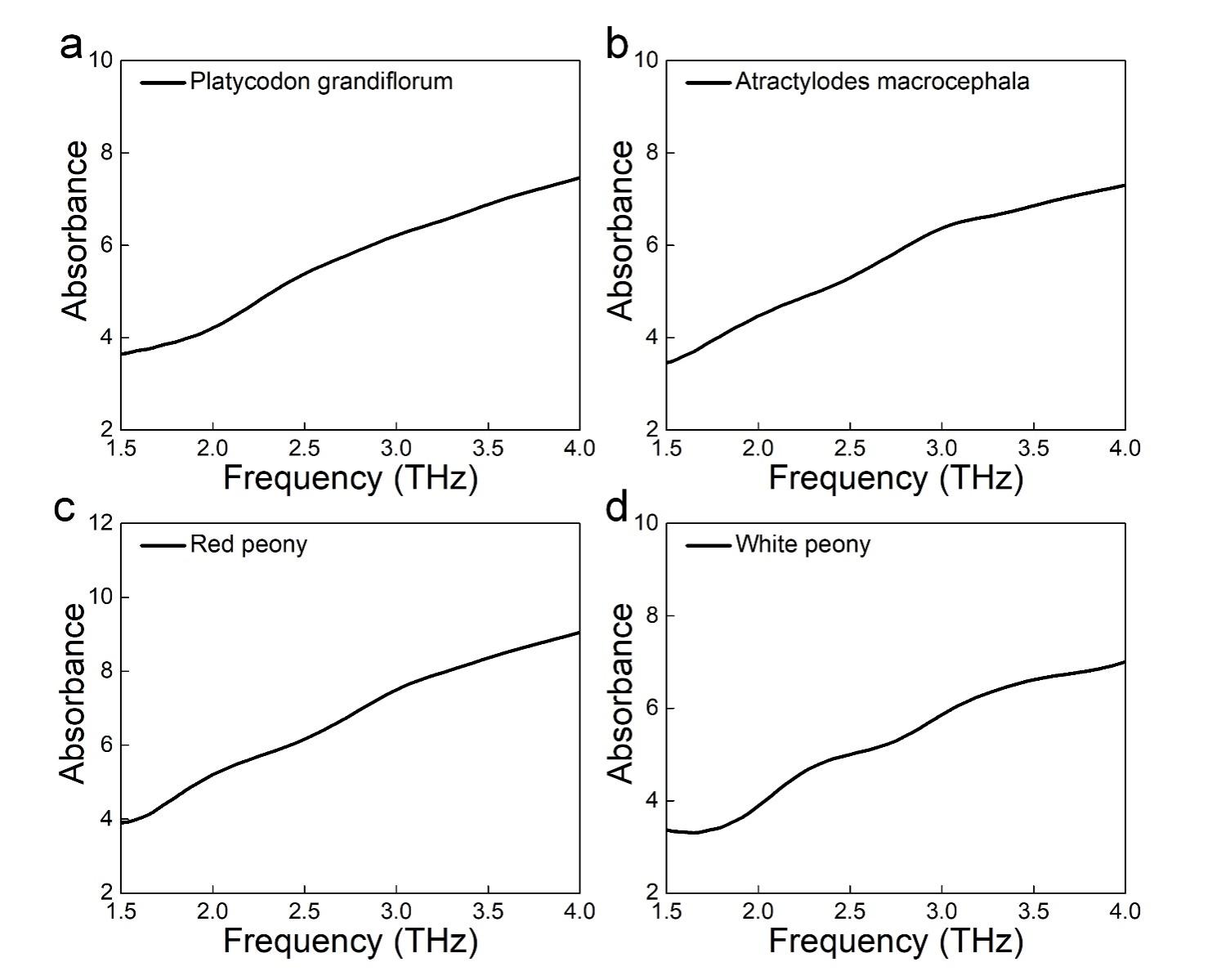


**Supplementary Figure 5.** **THz spectrum of** **non-Araliaceae herbs** (a) Platycodon grandiflorum. (b) Atractylodes macrocephala. (c) white peony. (d) red peony


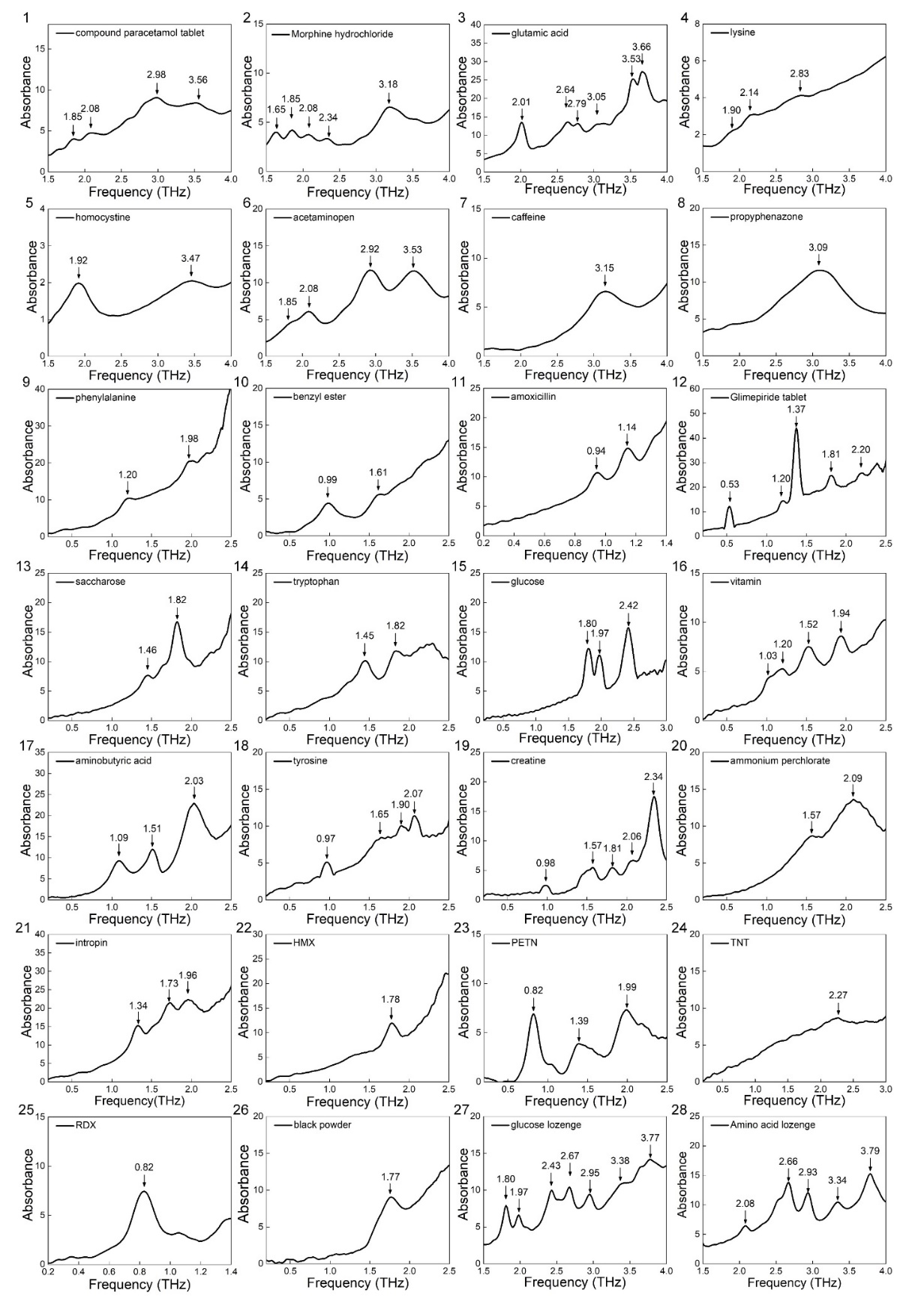


**Supplementary Figure 6.** **THz spectra of other 28 substances used for PCA analysis.** (1) compound paracetamol tablet. (2) Morphine hydrochloride. (3) glutamic acid. (4) lysine. (5) homocysteine. (6) acetaminophen. (7) caffeine. (8) propyphenazosne. (9) phenylalanine. (10) benzyl ester. (11) amoxicillin. (12) glimepiride tablet. (13) saccharose. (14) L-tryptophan. (15) glucose. (16) vitamin. (17) aminobutyric acid. (18) tyrosine. (19) creatine. (20) ammonium perchlorate. (21) intropin. (22) HMX(cyclotetramethylenete-tranitramine). (23) PETN. (24) TNT(Trinitrotoluene). (25) RDX(Hexogen). (26) black powder. (27) glucose lozenge. (28) amino acid lozenge.
