# Supplementary figures and images for: Terahertz Spectroscopy for Accurate Identification of Panax quinquefolium Basing on Nonconjugated 24(R)-Pseudoginsenoside F11
Source: Plant Phenomics. 2021 Jan 27;2021:6793457. doi: 10.34133/2021/6793457 (PMC8043154; doi:10.34133/2021/6793457)

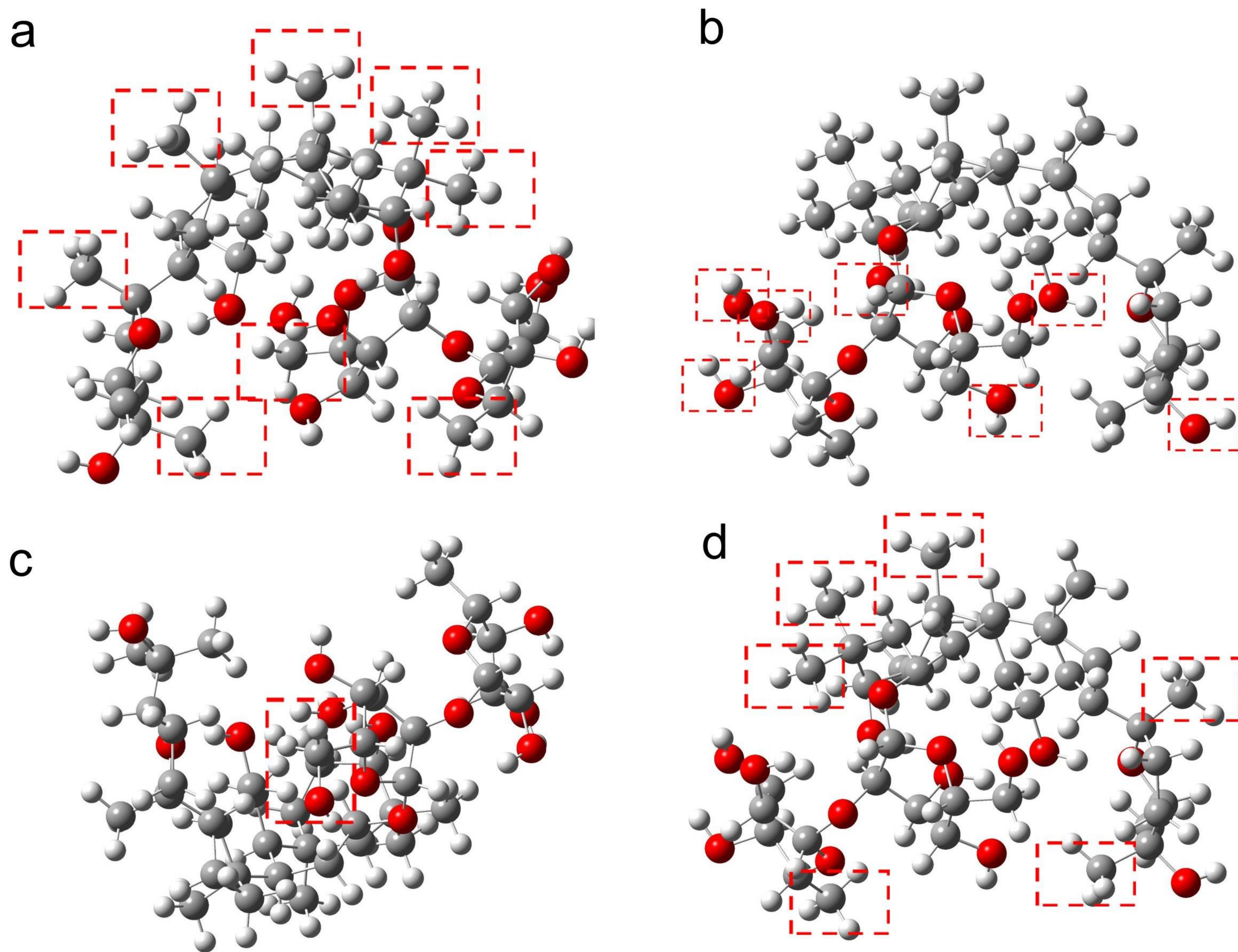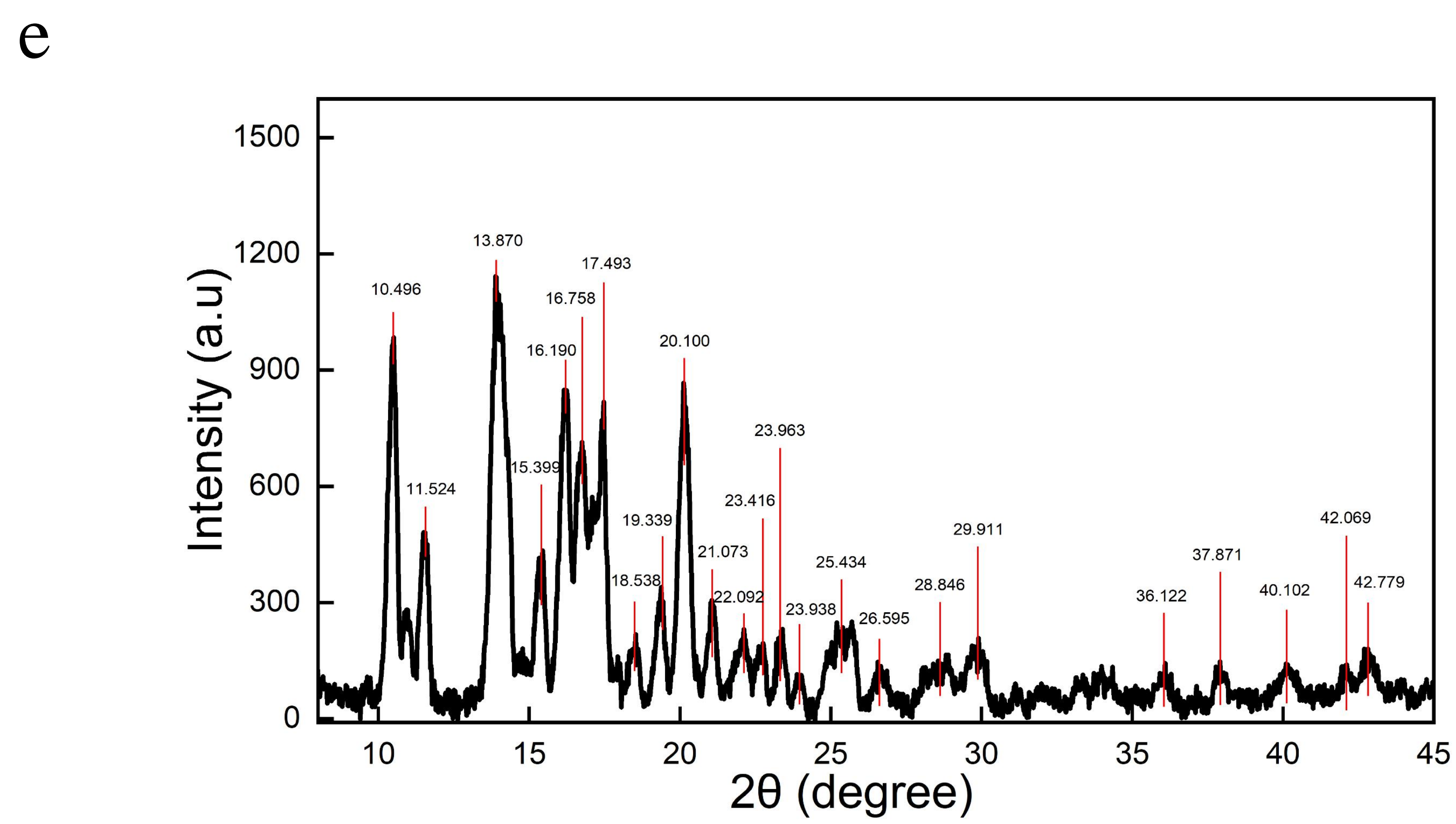

Supplement: Supplementary Materials — Supplementary 1 Principal component analysis method. Supplementary 2 Vibration mode analysis. Supplementary 3 Results of Panax quinquefolium MIR test. Supplementary 4 HPLC-QQQ-MS methods and results. Supplementary 5 THz spectra of substances used in PCA. [file 6793457.f1.zip › Supplementary Fig 1.pdf]

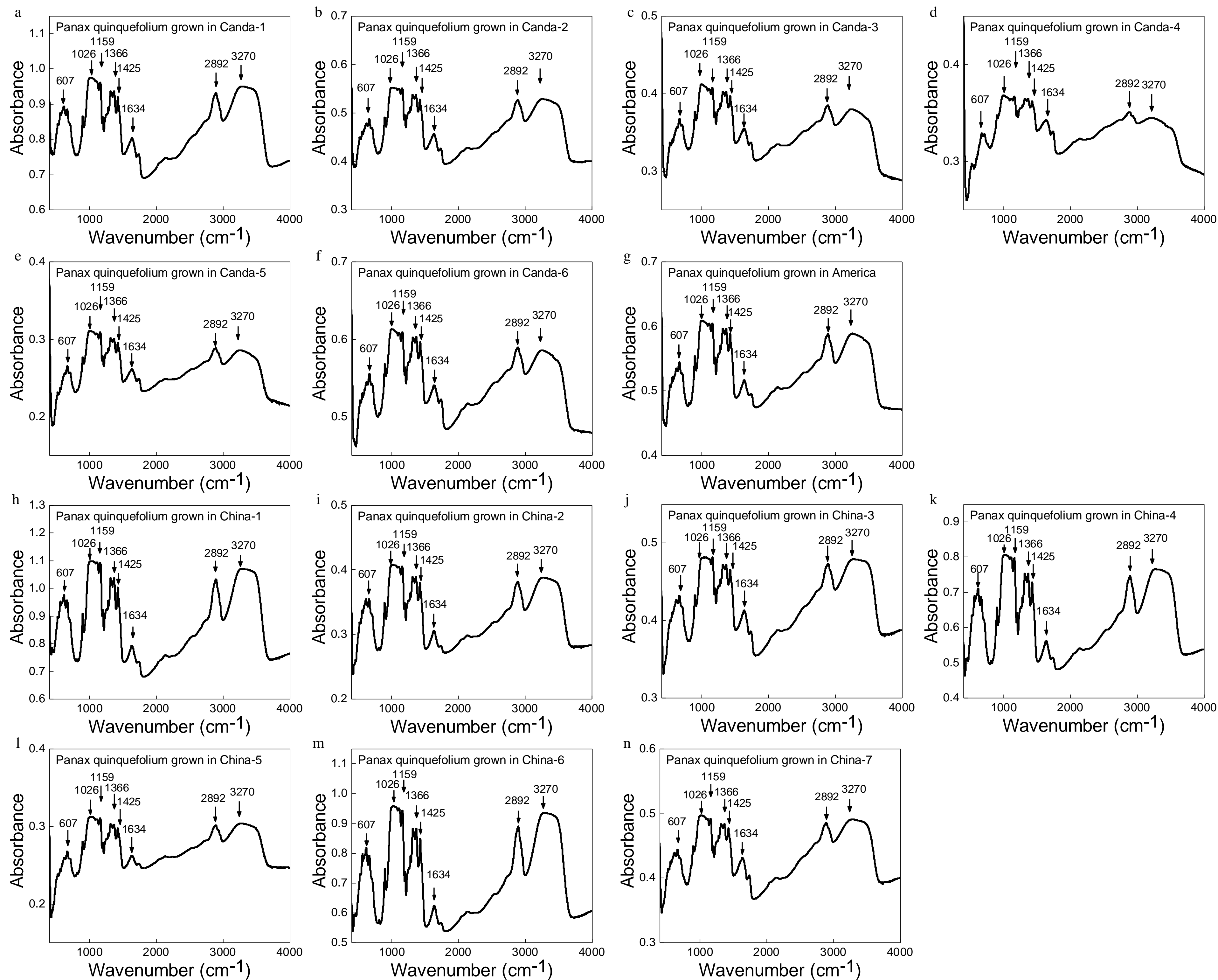

Supplement: Supplementary Materials — Supplementary 1 Principal component analysis method. Supplementary 2 Vibration mode analysis. Supplementary 3 Results of Panax quinquefolium MIR test. Supplementary 4 HPLC-QQQ-MS methods and results. Supplementary 5 THz spectra of substances used in PCA. [file 6793457.f1.zip › Supplementary Fig 2.pdf]

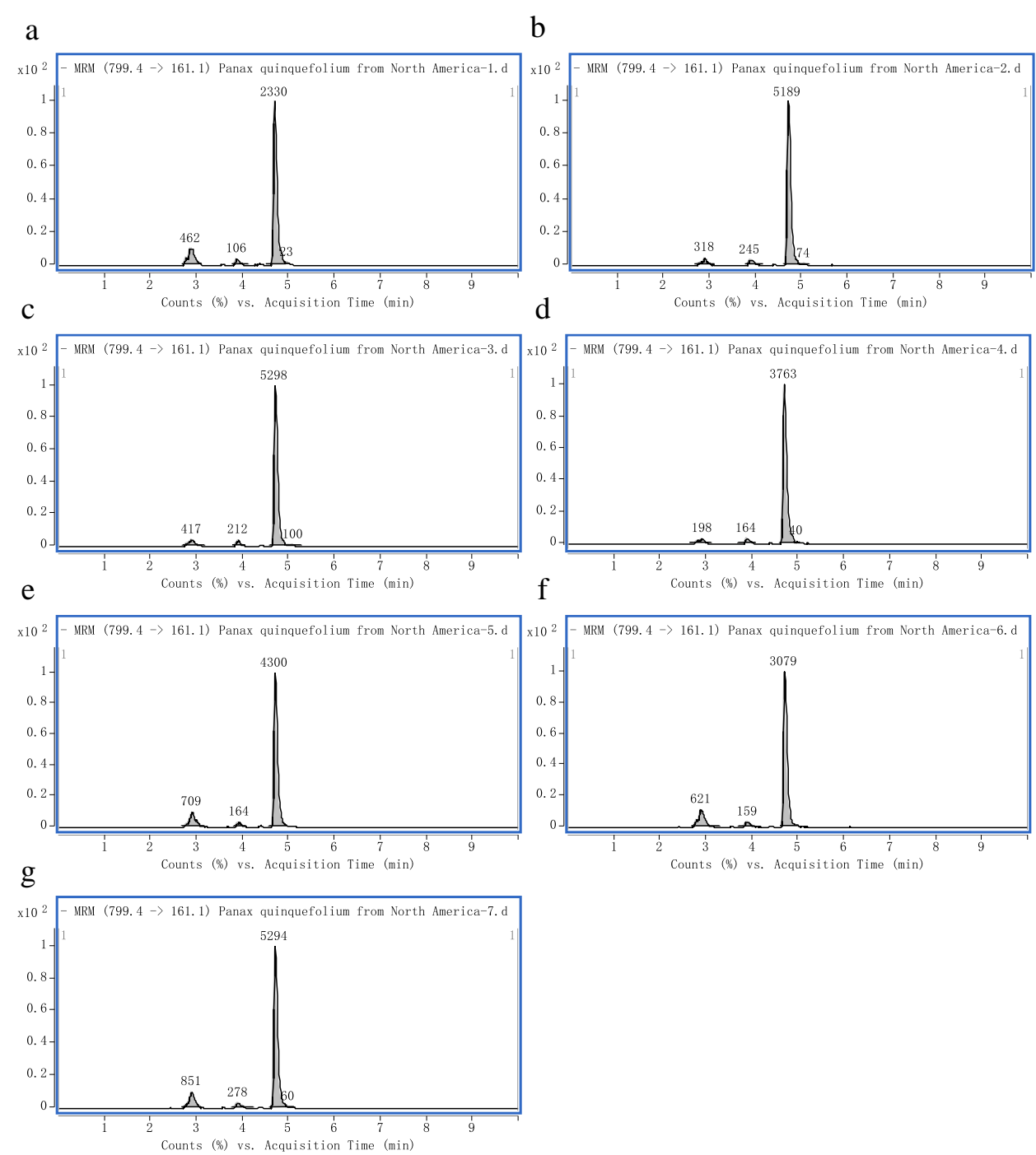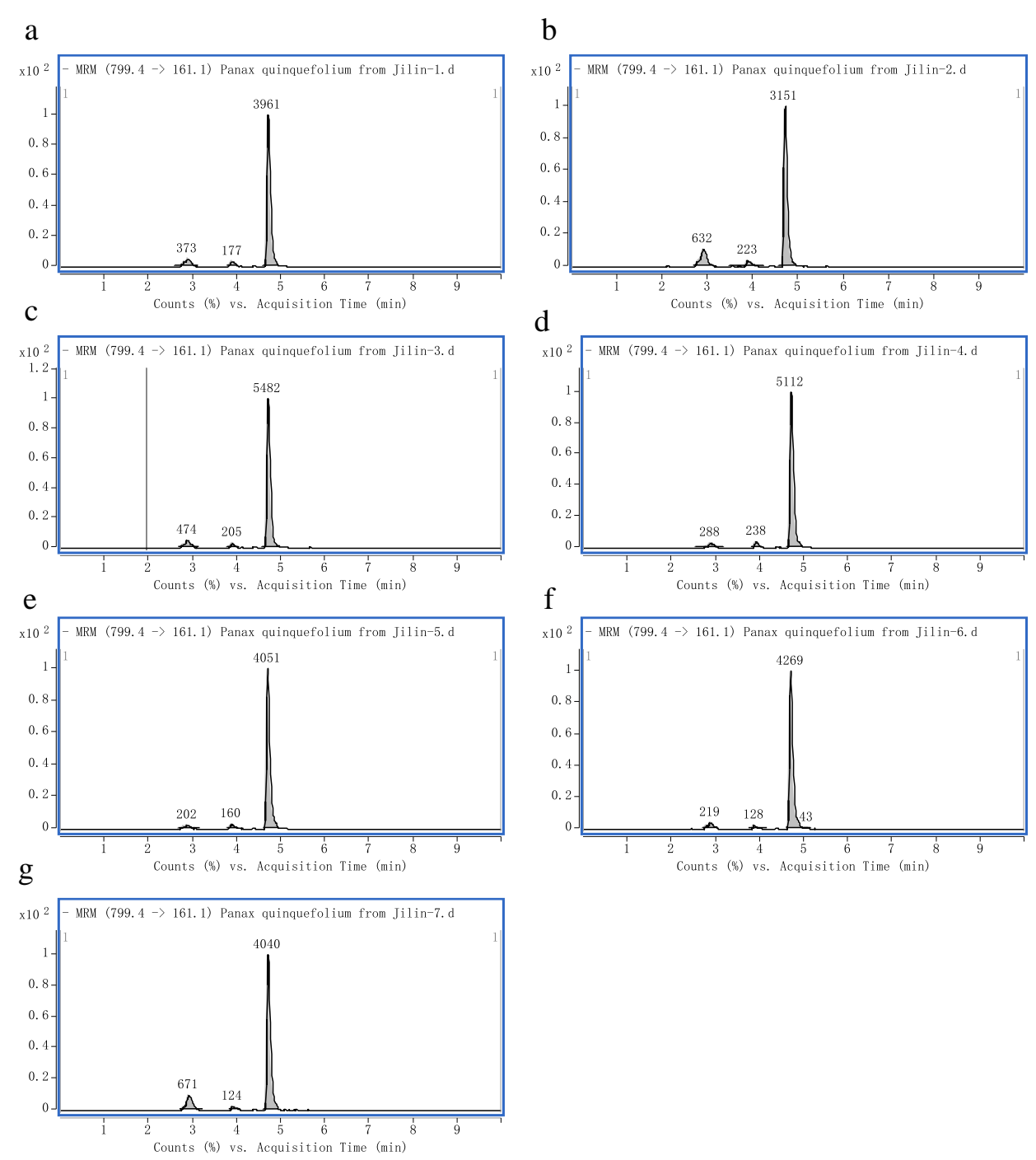

Supplement: Supplementary Materials — Supplementary 1 Principal component analysis method. Supplementary 2 Vibration mode analysis. Supplementary 3 Results of Panax quinquefolium MIR test. Supplementary 4 HPLC-QQQ-MS methods and results. Supplementary 5 THz spectra of substances used in PCA. [file 6793457.f1.zip › Supplementary Fig 3.pdf]

a

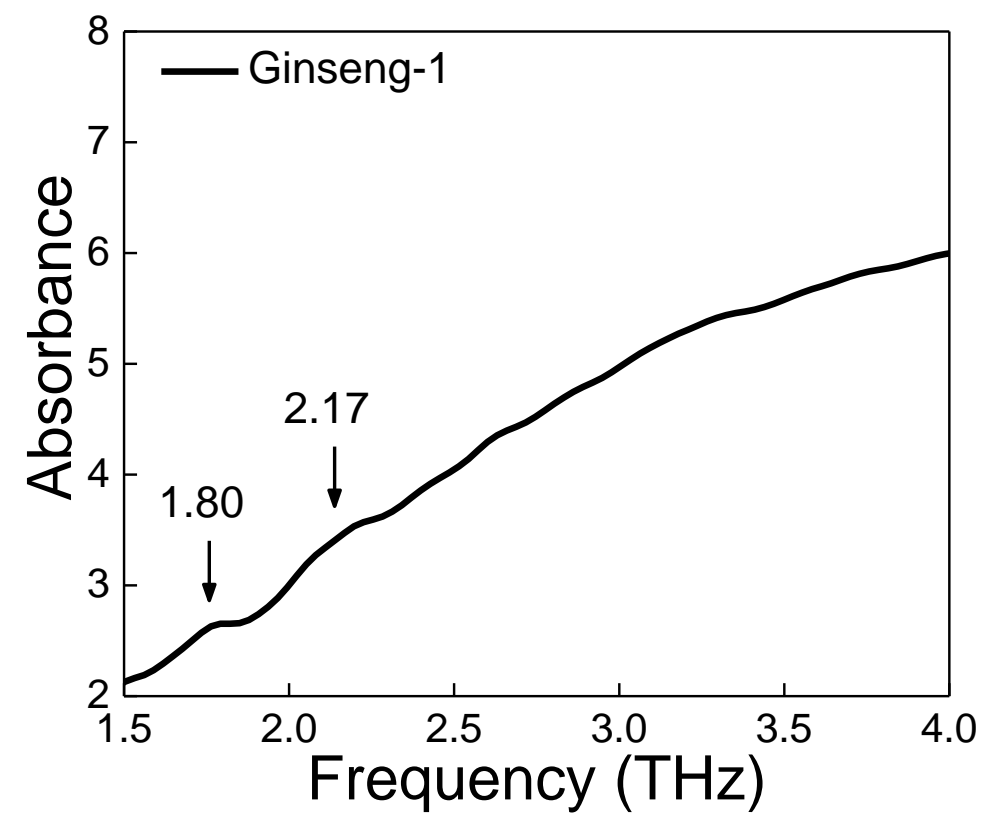

b

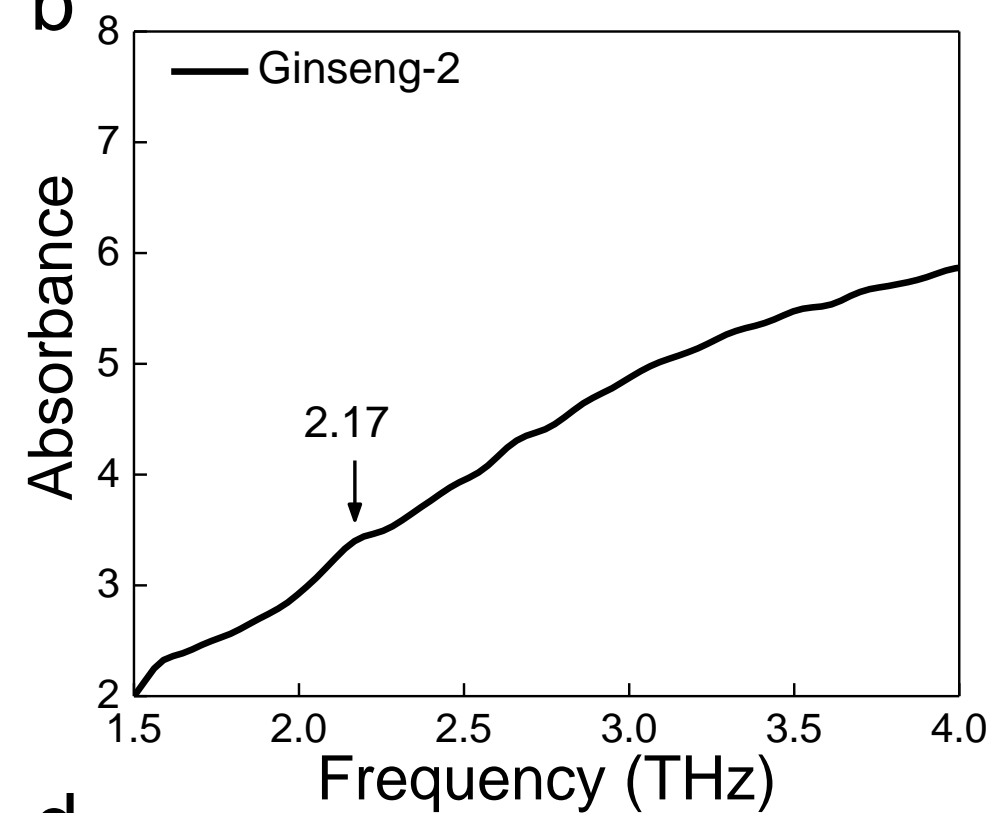

c

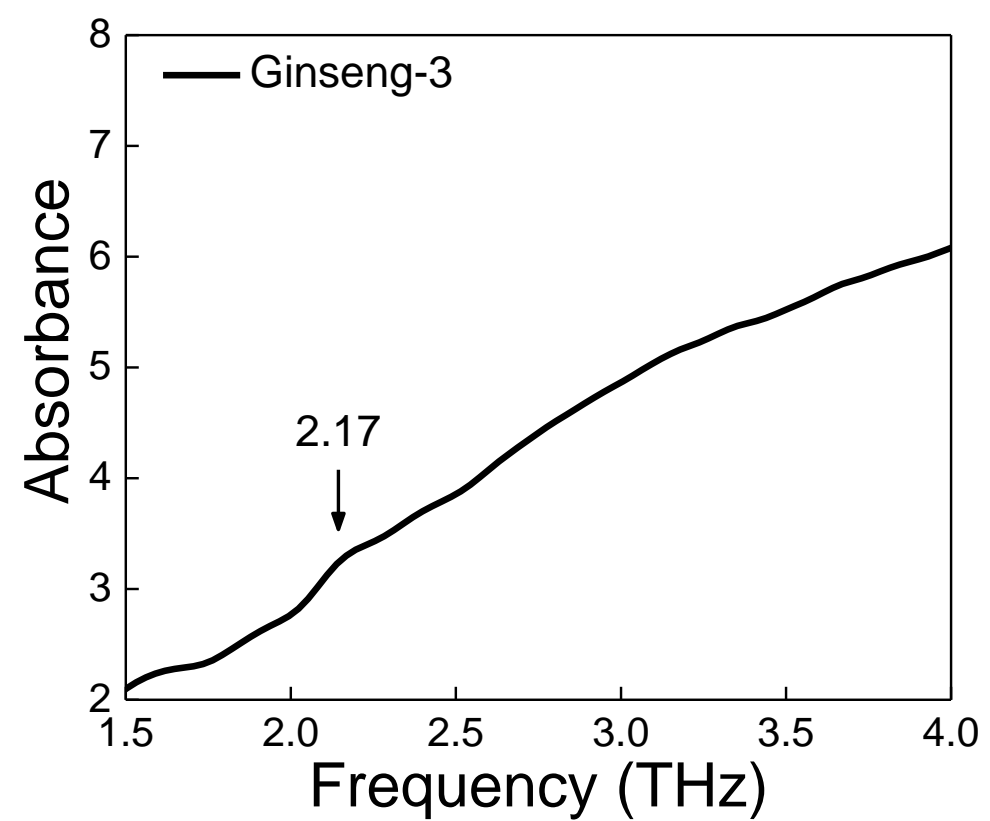

d

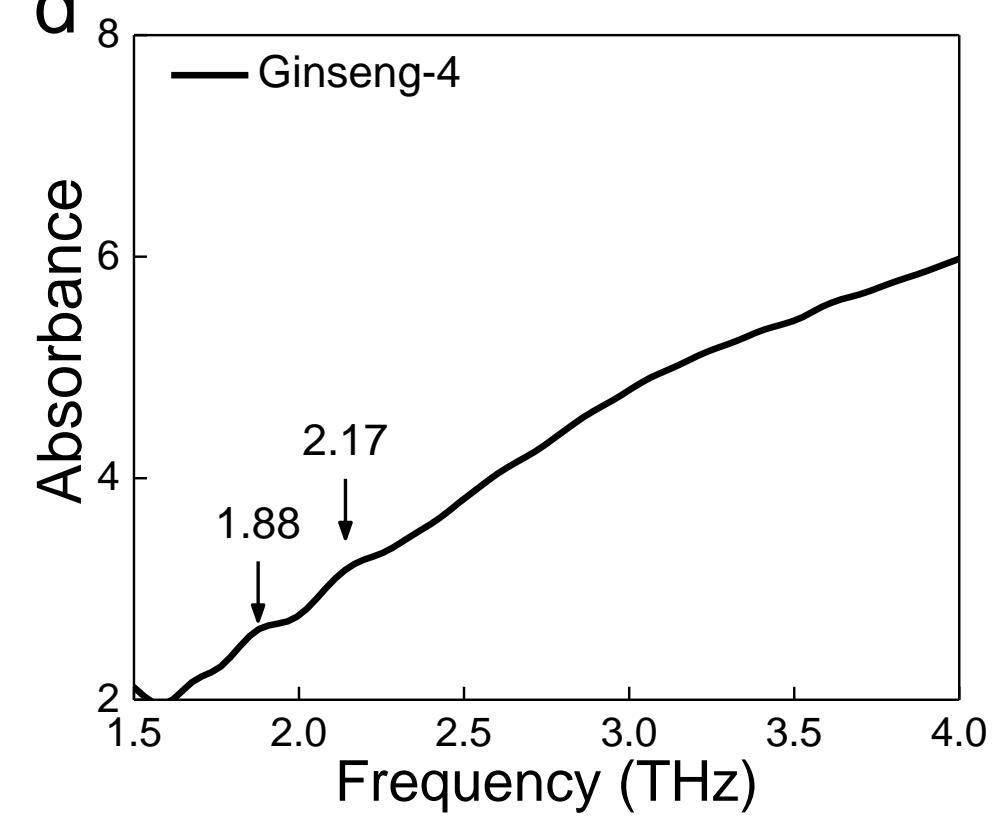

Supplement: Supplementary Materials — Supplementary 1 Principal component analysis method. Supplementary 2 Vibration mode analysis. Supplementary 3 Results of Panax quinquefolium MIR test. Supplementary 4 HPLC-QQQ-MS methods and results. Supplementary 5 THz spectra of substances used in PCA. [file 6793457.f1.zip › Supplementary Fig 4.pdf]

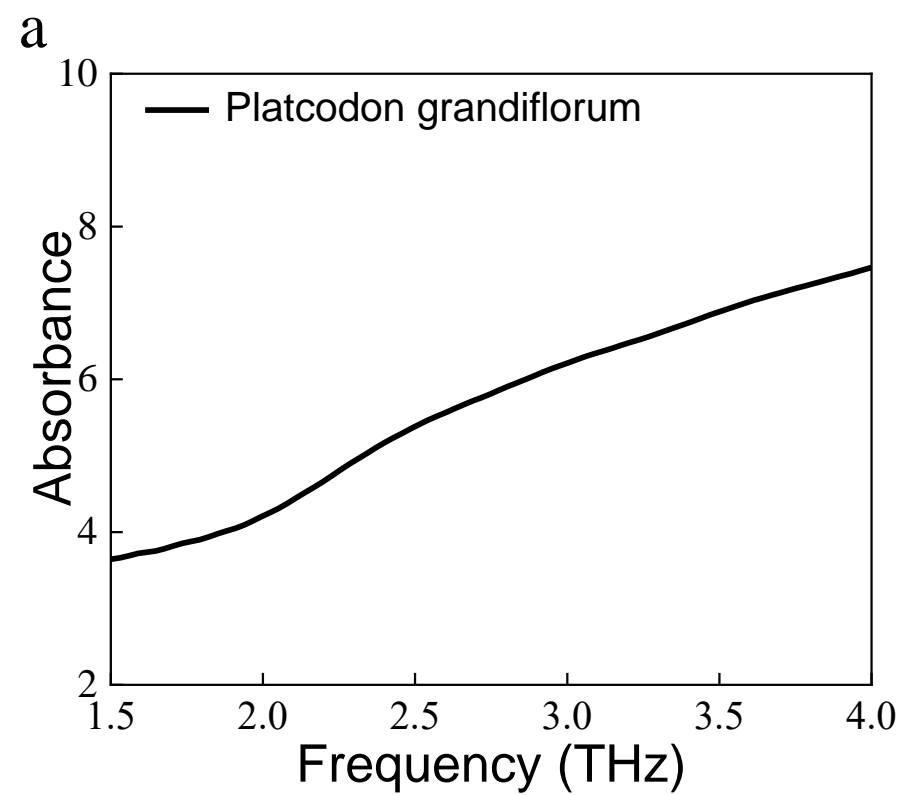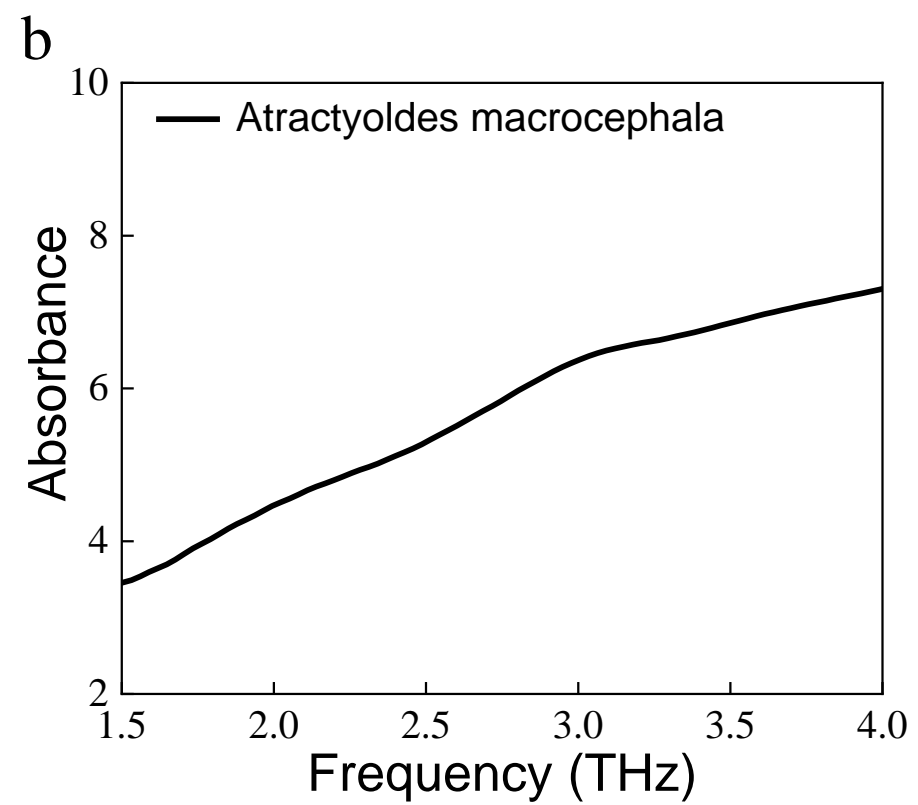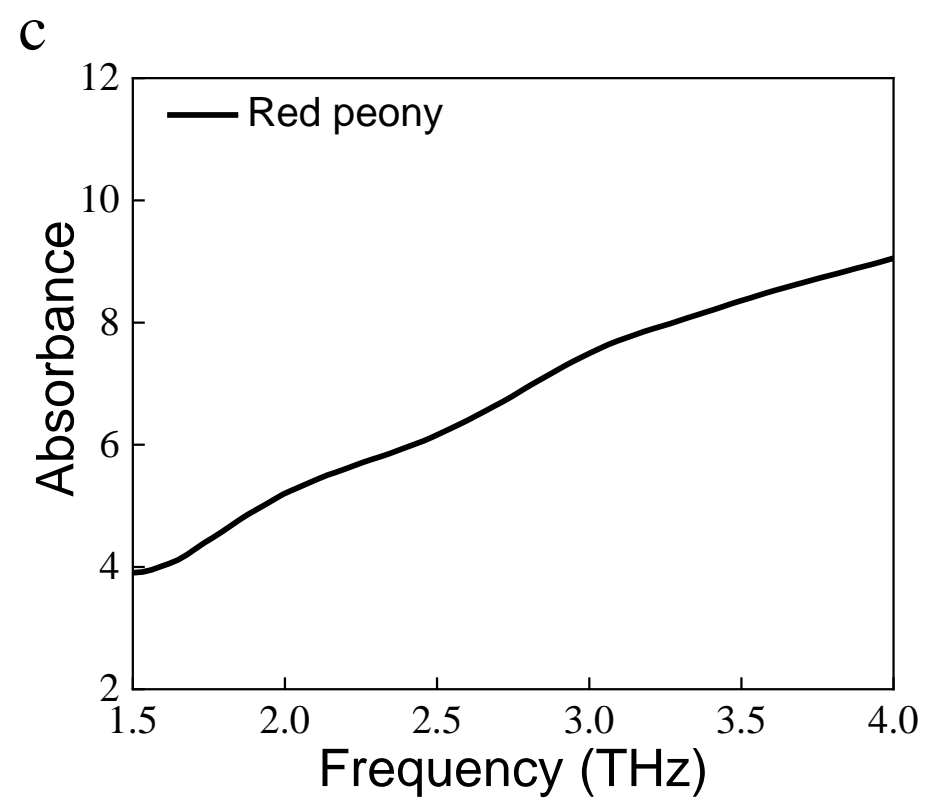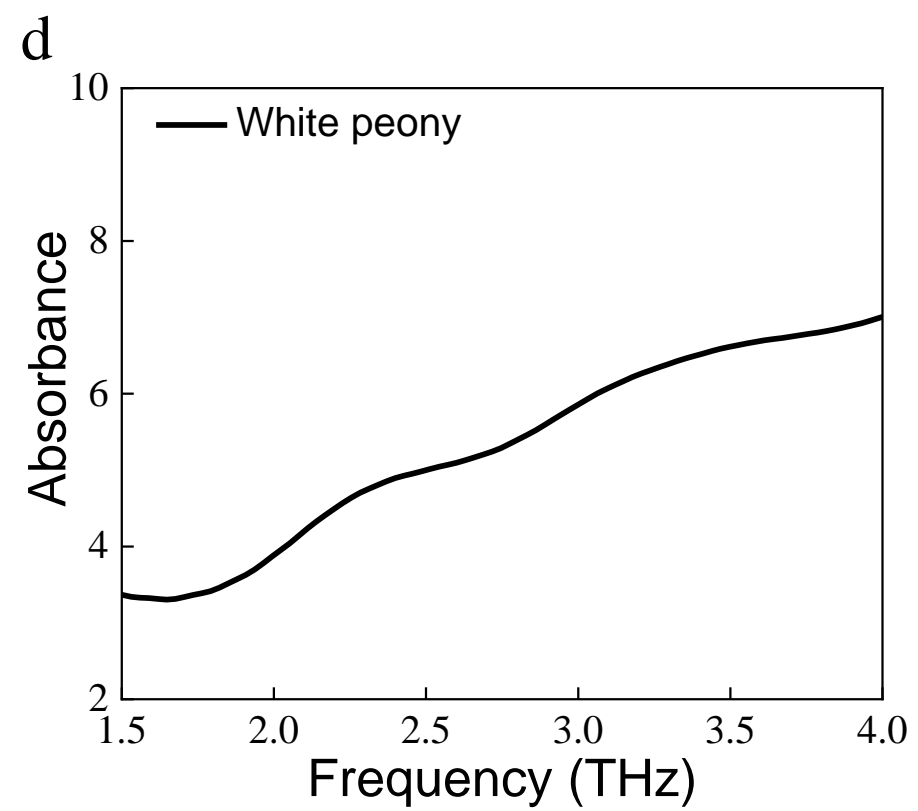

Supplement: Supplementary Materials — Supplementary 1 Principal component analysis method. Supplementary 2 Vibration mode analysis. Supplementary 3 Results of Panax quinquefolium MIR test. Supplementary 4 HPLC-QQQ-MS methods and results. Supplementary 5 THz spectra of substances used in PCA. [file 6793457.f1.zip › Supplementary Fig 5.pdf]

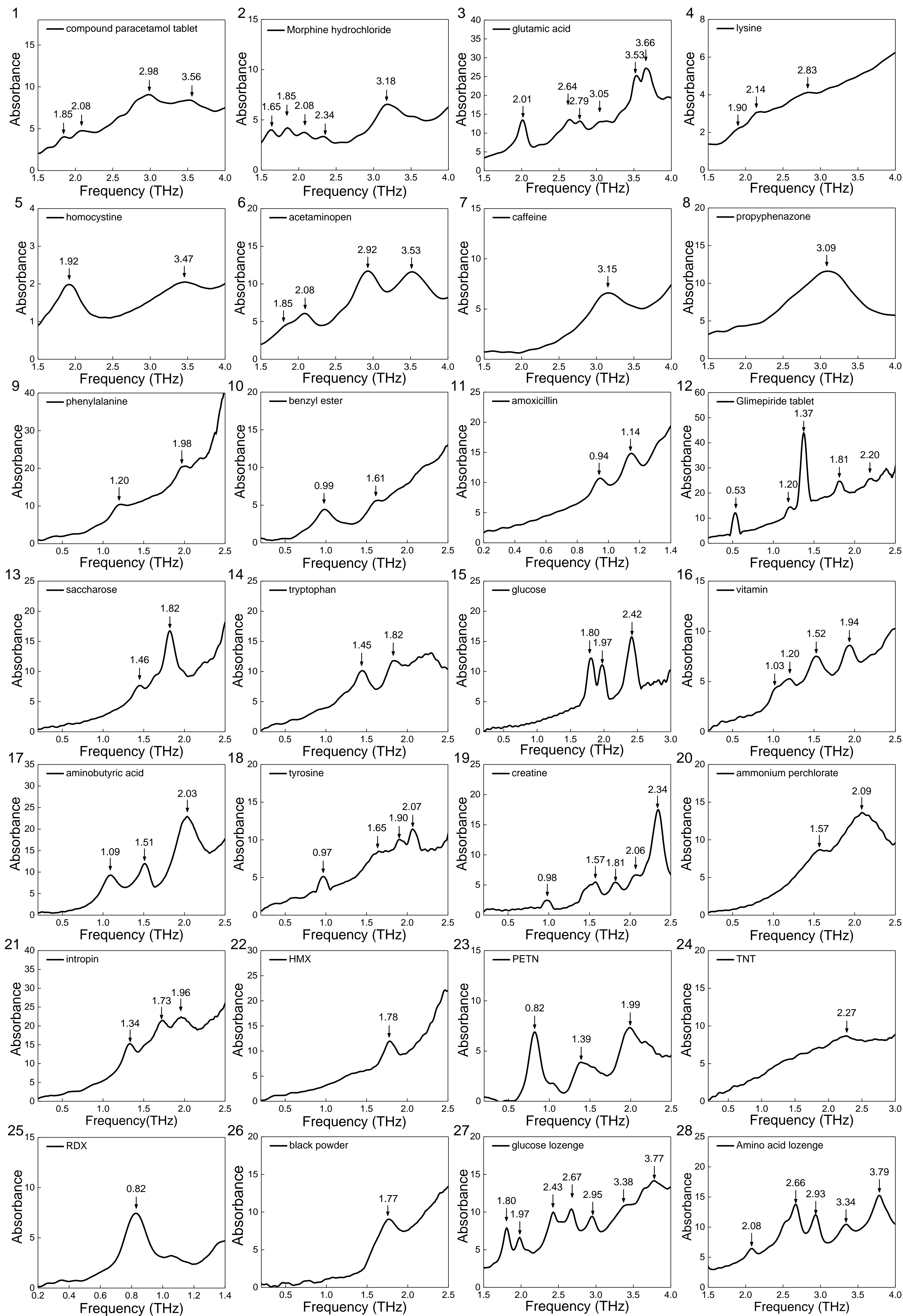

Supplement: Supplementary Materials — Supplementary 1 Principal component analysis method. Supplementary 2 Vibration mode analysis. Supplementary 3 Results of Panax quinquefolium MIR test. Supplementary 4 HPLC-QQQ-MS methods and results. Supplementary 5 THz spectra of substances used in PCA. [file 6793457.f1.zip › Supplementary Fig 6.pdf]
